# Age, experience, and homing performance in a long-distance migrating bird, the barn swallow

**DOI:** 10.1093/cz/zoaf080

**Published:** 2025-12-22

**Authors:** Piotr Matyjasiak

**Affiliations:** Museum and Institute of Zoology Polish Academy of Sciences, Wilcza 64, Warsaw PL-00-679, Poland

**Keywords:** age, barn swallow, experience, flight performance, *Hirundo rustica*, homing performance

## Abstract

Homing, the ability of animals to return to specific areas, is crucial for their survival and reproduction. It plays a fundamental role in shaping their spatial range by enabling them to maintain connections between distant critical habitats and optimize resource use. Homing performance, defined here as a combination of likelihood of return and homing time, is known to improve with repeated flights from the same starting location. However, the effect of age on this metric in adult migrant birds is still poorly investigated. I conducted an experiment to investigate whether age and experience gained from a previous homing flight along the same route influence the homing performance of adult male barn swallows *Hirundo rustica*. I found that the homing performance of male barn swallows increased consistently and significantly with their age. Homing performance was also significantly repeatable among individuals. Furthermore, return times from the same release location were significantly quicker on the second flight than on the first, regardless of whether the flights occurred in the same or following year. These findings suggest that, in at least one species of long-distance migratory passerine bird, homing performance improves with age and accumulated experience. To build on this knowledge, comprehensive research on the factors shaping homing performance in seasonally migratory bird species across diverse taxonomic groups is needed.

## Graphical Abstract

**Graphical Abstract:**
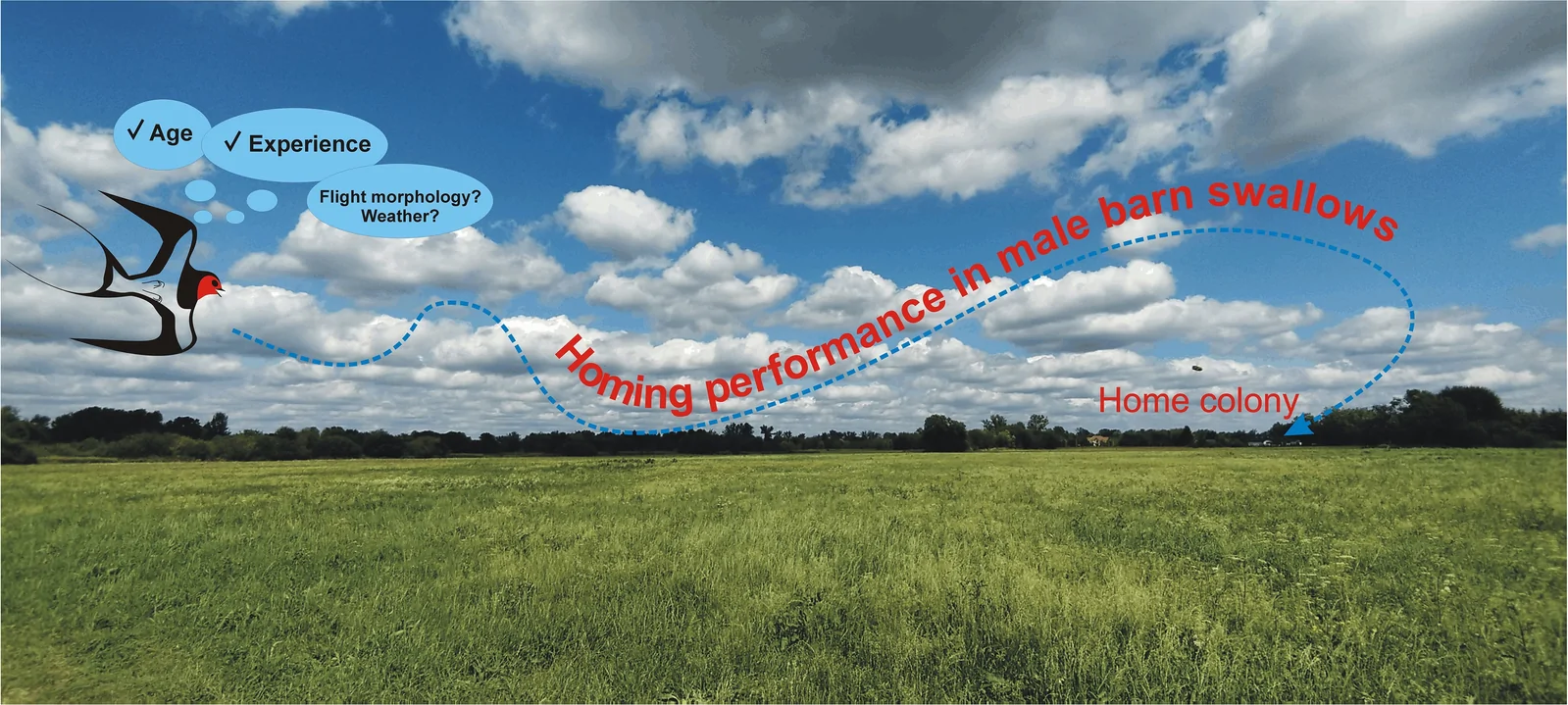

Homing is the crucial ability of animals to return to specific areas of importance for their survival and reproduction (Papi 1992). This behavior is shown across a diverse range of taxa (reviewed in Papi 1992; Åkesson et al. 2014), including social insects (Mandal 2018), fishes (Ward et al. 2013), amphibians (Pašukonis et al. 2014), reptiles (Zhang et al. 2024), mammals (Silovský et al. 2024), and, most notably, birds (Åkesson 2003). Homing is particularly significant for species that migrate seasonally or undertake foraging trips (Able 2001; Åkesson et al. 2014), as it enables precise return to their breeding or feeding sites. This ability plays a fundamental role in shaping the spatial range of mobile animals, allowing them to maintain connections between critical habitats over vast distances and optimize resource use (Jesmer et al. 2018; Lewis et al. 2021; Williams and Safi 2021).

Fast and successful homing relies on the integration of navigational capacities (e.g., visual, olfactory, and geomagnetic navigation skills; Wallraff 2004; Mann et al. 2014; Wiltschko and Wiltschko 2015; Zannoni et al. 2020) and locomotor abilities (e.g., flight performance; Dell'Ariccia et al. 2008; Taylor et al. 2017), which allow animals to efficiently orient themselves and traverse distances to return to their home location (Åkesson and Hedenström 2007; Åkesson et al. 2014). Although some navigational skills, such as the use of a geomagnetic compass, are innate, others—like the development of a navigational “map” based on geomagnetic, visual or olfactory cues—emerge as individuals mature, often in parallel with the progression of their locomotor abilities (Able 1991; Wiltschko 1991; Wallraff 2004; Åkesson et al. 2021). In mature individuals, these navigational skills and cognitive “maps” are further updated and refined through individual and/or social learning, which improves both their precision and the flexibility in applying various navigation skills and strategies (Åkesson et al. 2021).

In this study, I investigate the factors shaping the homing performance of birds during homing tests. Homing performance was defined as the hazard of an early return of the birds to their home location following displacement. Although most research on homing has focused on homing pigeons *Columba livia*, renowned for their excellent homing skills, this study examines a wild, migratory species, the Eurasian barn swallow *Hirundo rustica*. Specifically, I explore two factors—experience and age—that are likely to influence homing. Previous studies have consistently demonstrated that repeatedly releasing pigeons from the same locations improves their homing performance (Guilford and Biro 2014; Mehlhorn and Rehkaemper 2016; see Wiltschko 1991 for a review of earlier research). Such repeated flights enhance familiarity with surroundings and potential routes, thereby improving homing performance and return efficiency.

Research on homing pigeons of different ages, as well as comparisons between immature wild migratory birds (prior to their first autumn migration) and adults (after completing their first migration), have revealed substantial improvements in homing performance (Wiltschko 1991; Wiltschko and Wiltschko 2015; Åkesson et al. 2021). These improvements are thought to correlate with the development and refinement of individuals’ cognitive abilities, as discussed earlier. However, research on how homing performance develops as birds age and gain experience through successive episodes of seasonal migration, remains limited (Åkesson et al. 2021). To the best of my knowledge, this topic has been addressed in only one study: Sergio et al. (2014) reported age-related improvements in route efficiency and homing speed in migrating adult black kites *Milvus migrans*.

Other factors that shape homing performance include mating or reproductive status of individuals. For example, Mehlhorn and Rehkaemper (2016) reported that having a nest with eggs is an important motivator for homing pigeons to return home. Additionally, the degree of satiety has been shown to influence homing behavior: hungry pigeons, during homing trials, headed first to a food source and only then returned to the home loft (Blaser et al. 2013).

Barn swallows from European populations are long-distance migrants, wintering in sub-Saharan Africa (Turner 2006), with individuals from the population studied in this research migrating as far as southern Africa (Matyjasiak et al. 2023). Barn swallows are an ideal species for investigating homing behavior. They exhibit strong breeding philopatry: adults consistently return from migration to the same location and, in many cases, to the same breeding territory year after year. Swallows are known for their remarkable ability to successfully return after being displaced over long distances, even hundreds of kilometers, and for returning more quickly from sites where they have previously been released (Wojtusiak 1949; Nastase 1982). In addition, they nest in close proximity to humans, often within buildings, are not shy, and are very easy to observe.

I displaced male barn swallows of known age to the same release site twice within the same breeding season or twice across consecutive years, then recorded their returns to their breeding territories. I expected that, if familiarity with the release site and return route enhances homing performance, then males on their second flight would have a shorter time to return than on their first flight. Then, I hypothesized that, if the ability to correct for displacement improves with age and migratory experience, older males would return consistently faster than young males, irrespective of prior route familiarity.

## Materials and methods

### Study species and site

Barn swallows are small passerine birds (body mass: ca. 18 g, wing span: ca. 0.34 m), known for their strong flight abilities and for hunting insects in the air. During the breeding season, they are closely associated with human dwellings, typically nesting singly or in colonies on farm buildings and other human-made structures. In Europe, they usually nest in association with livestock farming (Møller 1994; Turner 2006). Barn swallow foraging trips during nestling feeding typically cover short distances—most are within approximately 1 km from the nest. Dispersal distances of young birds from the natal site (natal dispersal) are on average 11 km (range 0–261 km, depending on the study and region, with males moving shorter distances than females). Adult dispersal (breeding dispersal) averages about 1.1 km (range 0–45 km) (Turner 2006).

I studied the homing behavior of barn swallows in 2 breeding colonies near Warsaw, Poland (52°22′N, 20°52′E; see Matyjasiak et al. 2013 for description of the study area). The colonies are located in horse stable buildings and situated 1,200 m apart. During the 2016–2018 research period, these colonies consisted of 27–31 and 40–56 breeding pairs, respectively. The study population has been extensively ringed by frequent mist-netting as part of a long-term research project, which made it possible to accurately determine the age of the birds (Matyjasiak et al. 2013, 2024). Males hatched during previous calendar year were tagged as first year olds (1y-old; Euring number 5). The sex of barn swallows was determined at first capture, shortly after their spring arrival from Africa, based on differences in the shape of the cloacal region, or later by the presence (in females) or absence (in males) of a brood patch (Svensson 1992), or by observations of their behavior at the nests. To facilitate easy and precise individual recognition, all birds were marked with a combination of numbered metal and colored leg rings. Additionally, males participating in the homing tests were marked on the lower breast and belly feathers using a combination of dyes applied with garment-staining permanent markers. Only males were used, as the study was part of a larger project investigating various aspects of male performance. This approach also helped minimize disturbance to this strictly protected species.

### Homing experiment

I carried out homing experiments between May 20 and the end of June, during the first brood of barn swallows, over the years 2016–2018. The experiments were performed on sunny days, no rain, and winds no faster than 8 m/s. I calculated average air temperature, average wind speed and dominant wind direction for each homing trial, using publicly available data from a weather station in Legionowo (https://hydro.imgw.pl/#/), located 6.5 km from swallow colonies under study and 52 km from the release site.

Male swallows were captured between 08:00 and 10:00 AM, allowing them time to feed and maintain themselves before homing test. When many barn swallows were present in their territories inside the stable, the gate and all window openings were closed. Mist nets were then set up across the stable corridor, at the main entrance, and at selected windows. Once in place, the entrances were reopened, and the birds were gently guided out to fly into the nets. The trapping lasted from 0.5 to 1 h.

Trapped males of known age were randomly assigned to two groups. The first group were to underwent their second homing flight in the same year, while birds from the second group were to complete their second flight the following year. Males were placed in transport containers (cardboard xerographic paper boxes with perforated walls, five birds per box) and transported by car to the release site, a journey lasting 1.2 h. The release site was located near Wyszogród, on a high escarpment of the right bank of the Vistula River (52^o^23′N, 20^o^11′E, home direction = 93^°^ E), approximately 47.8 and 48.9 km from the two home breeding colonies, respectively. The release began 2 h after trapping was completed and took place between 11:00 AM and 1:00 PM. All birds were released individually. The release interval was determined based on pilot observations using binoculars (10 × 40) to record the departure times of male barn swallows (*n* = 24) from a 500-m zone surrounding the release site, delineated by natural landmarks such as trees and bushes. Data from 4 birds that flew low and disappeared prematurely behind vegetation were excluded. For the remaining individuals, the time to leave the release zone averaged (mean ± SD) 1.6 ± 0.5 min (range 0.8–3.2 min, *n* = 20). The release interval was set at 6 min—twice the maximum observed departure time—to minimize the risk of birds returning in pairs or groups.

Two observers (including me) continuously monitored the territories of the test males by walking along the stable corridor. Special care was taken to prevent interference with the focal males’ nests by neighboring or unpaired males. Observations continued for 6.0 h after the first male was released. Return observations confirmed that all focal males returned individually, ensuring that time-to-return measurements reflect individual performance rather than group guidance. The fate of males that did not return during the observation period was tracked during routine observations performed daily at the breeding colonies. Binoculars (Vortex Viper, 10 × 40) and scopes (Ecotone SP-80, 20–60 × 80, focus down to 6 m) were used to help identify returning males.

### Statistical analysis

My aim was to investigate how male age and experience influence homing in male barn swallows. To achieve this, I analyzed homing performance via two metrics: homing success (return vs. no return during the observation period) and homing time (duration to return). Homing time serves as a precise, quantitative metric that captures variation in individual locomotor performance, navigational ability, and motivation among birds that successfully return. In contrast, failure to return within the observation window reflects potential limitations in these same traits, and may indicate a threshold below which homing is unsuccessful. Together, these two measures offer complementary insights into the factors that shape variation in homing performance and may reflect an individual's ability to navigate and traverse distances effectively (Papi 1992). It must be acknowledged that pure navigational efficiency (e.g., route efficiency, defined as beeline distance divided by actual distance) cannot be assessed without GPS-derived route metrics (Steiner et al. 2000; Biro et al. 2006; Dell'Ariccia et al. 2009). However, studies on homing pigeons have shown that shorter homing times might be correlated with higher route efficiency (Dell'Ariccia et al. 2008, 2009; Mehlhorn and Rehkaemper 2016). Although homing time alone is influenced by motivational factors such as waiting time, hunger, mid-journey pauses or detours (e.g., Dell'Ariccia et al. 2008, 2009; Blaser et al. 2013), standardized release protocol used in the present study, controlled release distance, and robust sample sizes minimize such confounding effects.

I modeled homing times using a Cox proportional hazards framework, in which each bird's return time is the survival time, and non-returns within the observation window are treated as right-censored events (status = 0). A return event (status = 1) occurs when the bird arrives back on its territory during the observation period. This approach allowed me to account for the fact that some males could have completed their homing flight within the observation window if given more time. Predictor variables (e.g., age, experience) enter the hazard function such that a higher hazard ratio corresponds to a greater instantaneous probability of return—that is, higher homing performance under standardized conditions (including any pauses or detours), rather than pure navigational ability (i.e., route efficiency).

I relied on mixed-effects Cox proportional hazards models using the “coxme” v.2.2.22 and “survival” v.3.8.3 packages (Therneau 2024a, 2024b) in R (R Core Team 2024). I adopted a hypothesis-driven modeling approach following the guidelines by Harrison et al. (2018) . First, I built an initial Cox mixed-effects model including two empirically justified fixed predictors (a priori hypotheses): male “age” (3-level factor; “1y-old” [reference level], “2y-old,” and “older” males), and “repeat type” (3-level factor: “first flight” [reference level], “within year,” and “among year’). Among the two a priori predictors, “age” captures the improvement in homing performance that males achieve as they age and develop their navigational skills through successive seasonal migration events, while “repeat type” reflects improvements in homing performance that occur as males gain experience from being released at the same release site—either within the same year (“within year”) or across consecutive years (“among year”). Homing time (in minutes) was a response variable. I categorized each homing flight with one of two status values: 1—if the male returned during the observation period, and 0—if the male returned later (censored observation). The males’ identity (nested within the “colony,” 2-level factor: “colony 1,” “colony 2”) was included in the model as a random intercept to account for the fact that many of the homing tests were repeated on the same individuals.

Next, additional predictors, including morphological traits and weather conditions, were evaluated individually through screening models. The additional predictors included “breeding stage” (expressed as the number of days since the first egg was laid) and “release order” (to control for waiting time for the homing flight) as discrete numeric variables, and an interaction term between “age” and “breeding stage”. Male “wing aspect ratio,” “tail length,” “keel length,” and “body weight” were included as continuous covariates to control for variation in individual flight morphology, body condition and body size. “Mean temperature,” “dominant wind direction” and “mean wind speed” were included to control for weather conditions during homing flights. Each additional predictor was added one at a time to the initial Cox mixed-effects model to test its independent association with homing time. Model performance was compared using the corrected Akaike Information Criterion (AICc; Burnham and Anderson 2002). Details of the screening models and the methods used for their comparison are provided in the Supplementary Table S1. Predictors whose inclusion improved model fit by more than 2 AICc units (ΔAICc ≤ −2) relative to the initial model were retained for further consideration.

All numeric predictors were scaled to mean = 0 and standard deviation SD = 1 before analysis. I used the Schoenfeld test (the “cox.zph” function in the “survival” package Therneau 2024a) to make sure that the proportionality of hazards assumption of the Cox regression is satisfied. To assess multicollinearity among the explanatory variables, I calculated variance inflation factors (VIFs). VIF values were obtained using the “vif” function from the “car” v.3.1.3 package (Fox and Weisberg 2019), applied to a global linear model with “lm” function from the “base” R, including all predictor variables. All VIF values were <2.0, indicating low levels of collinearity (Zuur et al. 2009).

I estimated the repeatability (intra-class correlation coefficient, ICC) of homing times across trials repeated on the same males using the “rpt” function from the “rptR” v.0.9.23 package (Stoffel et al. 2017), with Gaussian error structure. The linear mixed-effects model included male identity as a random effect and controlled for the fixed effects of the “age” and “repeat type” categories. I estimated confidence intervals for the repeatability estimate using 1,000 parametric bootstrap samples.

Based on the results of the screening stage (see Supplementary Table S1), I fitted a final mixed-effects Cox model that included the a priori predictors (‘age” and “repeat type’) as well as an additional predictor, “temperature”. I compared the initial and final “coxme” models using ΔAICc and the likelihood-ratio (LRT) test. I also evaluated the predictive improvement using the concordance index, which was computed from full linear predictors (fixed + random effects), with standard errors (SE) estimated using 1,000 nonparametric bootstraps. To validate the robustness of the results, I fitted analogous initial and final Cox proportional hazard models using the “coxph” function with gamma-distributed frailty term for male identity.

To visualize the influence of significant predictors on male homing performance, I used the “survfit” function from the “survival” package to estimate survival curves, with the survival function “proportion of males that have not yet finished their homing flight” as the dependent variable and “homing flight duration (minutes)” as the independent variable.

I assessed the effect of the experimental treatment on reproductive success by comparing the total seasonal number of fledglings between males involved in homing trials and untested individuals. I used a mixed-effects generalized linear model with a Poisson distribution (“glmer” function from the “lme4” v.1.1.36 package) for the comparison. The model also accounted for the males’ age, colony, and the year of the study. The males’ identity was included in the model as a random intercept. All models were fitted using the R v.4.4.2. environment (R Core Team 2024).

## Results

I conducted homing experiment on 97 male barn swallows. Of these, 54 males were tested twice within the same year, 20 were tested twice across two consecutive years, and 23 males were tested only once in one year. In total, I carried out *n* = 171 homing tests. In 112 homing tests (65.5%), males returned to their home territories during the observation period, while in 59 (34.5%) they returned after the end of the observation period, the next day at the latest. All males returned from homing flights.

The initial mixed-effects Cox model, which incorporated male identity as a random intercept, revealed significant effects of the two a priori predictors—“age” and “homing flight”—on time to homing flight completion (Table 1). As indicated by higher hazard ratios, older males and those flying the homing flight for the second time exhibited shorter return durations. Moreover, I found a significant estimate for the random intercept variance (Table 1), indicating significant differences in the baseline hazard of early return from homing flight between individual males. This means that homing performance is significantly repeatable among male barn swallows: some individuals consistently achieved shorter homing times, while others consistently recorded longer homing times, regardless of age or whether it was the first or second homing trial.

**Table 1:** Results of the initial mixed-effects Cox proportional hazard model—incorporating male ID as a random intercept—that analyzed the effects of two a priori predictors: “age” group and “repeat type” category (homing flight repeated “within year” or repeated “among year”) on the hazard of early return from homing flight in male barn swallows.

Fixed effects	Beta ± SE	HR	95% CI	*Z*	*P*
Age:2y-old males	1.365 ± 0.381	3.915	1.855 to 8.263	3.58	< 0.001
Age: older males	1.687 ± 0.449	5.402	2.240 to 13.026	3.76	< 0.001
Repeat type: within year	1.655 ± 0.253	5.232	3.186 to 8.594	6.54	< 0.001
Repeat type: among year	1.977 ± 0.424	7.221	3.147 to 16.569	4.67	< 0.001
**Random effect**	**Variance ± SE**	**95% CI**	**χ^2^**	**df**	***P***
Male: intercept	1.953 ± 0.141	1.500 to 2.648	263.24	66.5	< 0.001
**Model fit**	**Value ± SE**	—	—	—	
Concordance index	0.892 ± 0.017	—	—	—	

Reported are: regression coefficient (beta) with standard error (SE), hazard ratio (HR) with 95% confidence interval (CI), variance for the random intercept of male ID with SE and 95% CI, significance, and model concordance with SE. A HR > 1 indicates an increased hazard of earlier return from homing flight. Concordance represents the proportion of all usable pairs of males where predictions and observed outcomes are concordant, thus measuring the model's discriminative ability (values closer to 1 indicate better prediction).

The repeatability analysis confirmed the latter result. The ICC was estimated at *R* = 0.442 (± 0.093 SE, 0.259–0.604 CI, Likelihood Ratio Test: *P* < 0.001), indicating significant but relatively low repeatability of homing performance across repeated homing flights by the same male barn swallows. Approximately 44% of the total variation in return time was attributable to differences between males, suggesting a modest but consistent individual component in homing performance.

Among the screened additional predictors (Supplementary Table S1), only “mean temperature” met the selection threshold (ΔAICc ≤ −2 relative to the initial model). This predictor was included in the final Cox model.

The final Cox mixed-effects model revealed that older “age,” repeated homing flights (“repeat type”), and higher mean temperatures during the homing trial were associated with an increased hazard of earlier homing flight completion, i.e., faster return time and higher homing performance (Table 2; Figs. 1 and 2). The final model provided a substantially better fit to the data than the initial model, as indicated by a lower AICc value (ΔAICc < −2 relative to the initial model; Supplementary Table S1) and a significant likelihood ratio test (*LRT* = 4.26, *P* = 0.039). Although the final model exhibited a slightly higher concordance index (Tables 1 and 2), the difference was within the standard error, suggesting negligible improvement in predictive performance. A hazard ratio analysis showed that, compared with 1y-old males, 2y-old males were 4× better and older males were 6× better at returning from homing flight early (Table 2; Fig. 1). For the second homing flight, the hazard of the males returning to the territory early was 5 to 6× higher than the first time, when the homing flight was repeated within the same year or between two consecutive years, respectively (Table 2; Fig. 2). The survival curves, which illustrate the probability of male barn swallows continuing their homing flights and were estimated separately for each combination of “age” group and “repeat type” category, are shown in Supplementary Figure S1. For the effect of “mean temperature,” a 1 SD (4.0 °C) increase in this metric was associated with a 31% increase in the hazard of an early return from the homing flight (Table 2, Fig. 3). The direction, significance, and estimated effect sizes of the fixed effects remained consistent across both the “coxme” and “coxph” models, supporting the robustness of the results regardless of the random-effects parameterization (Tables 1 and 2, Supplementary Table S2 and S3).

**Figure 1:**
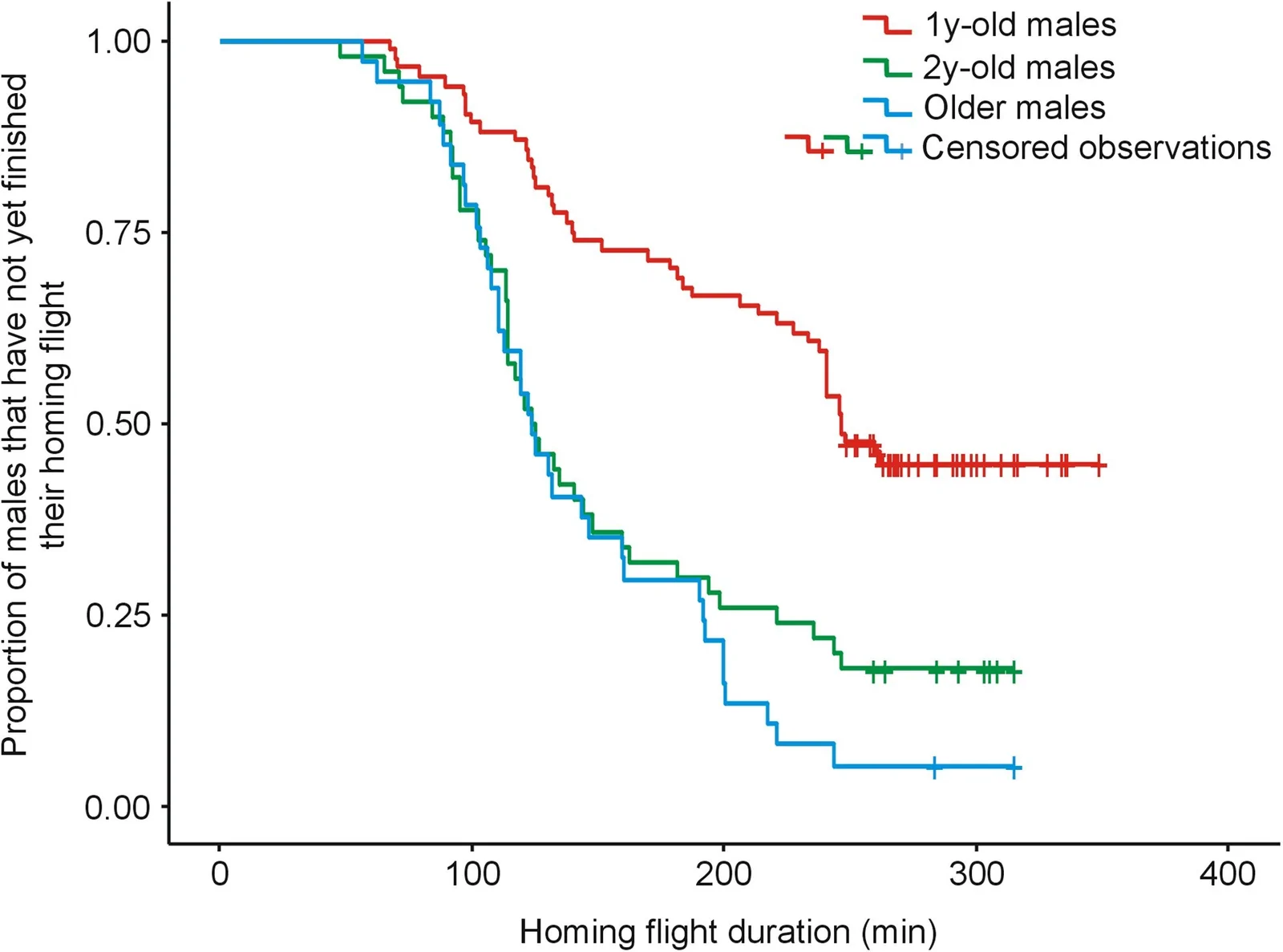
Survival curves illustrating the probability of continuing the homing flight as a function of male barn swallows’ age group. Survival curves for two levels of the predictor “age” (“2y-old” and “older” males) are contrasted with the curve for the reference level (“1y-old” males). “1y-old” males are 1-year-old males (males that hatched during previous calendar year). “2y-old” males are 2-year-old males (or males in their third calendar year of life). Lower survival curves indicate faster completion times for the homing flight. Older males tended to complete the homing flight more quickly, as reflected by the steeper decline in survival probability over time.

**Figure 2:**
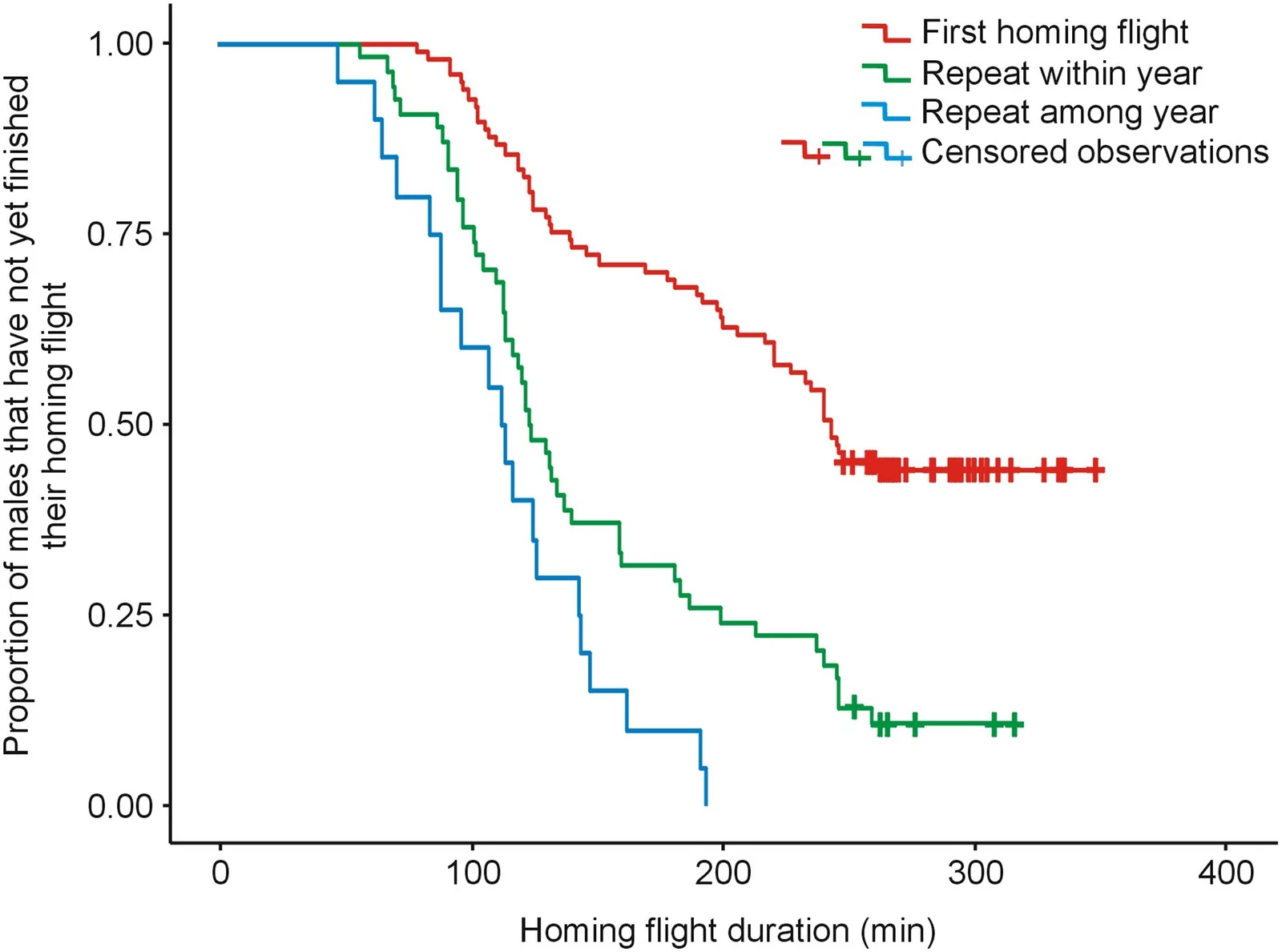
Survival curves illustrating the probability of continuing the homing flight as a function of male barn swallows’ homing trial group. The survival curves for two levels of the predictor “repeat type” (homing flight repeated “within year” or repeated “among year”) are contrasted with the curve for the reference level (“first flight”). Lower survival curves indicate faster completion times for the homing flight. During their second homing trial, whether within or among years, males completed their return flight more quickly than during their first attempt. This was reflected in a steeper decline in survival probability over time.

**Figure 3:**
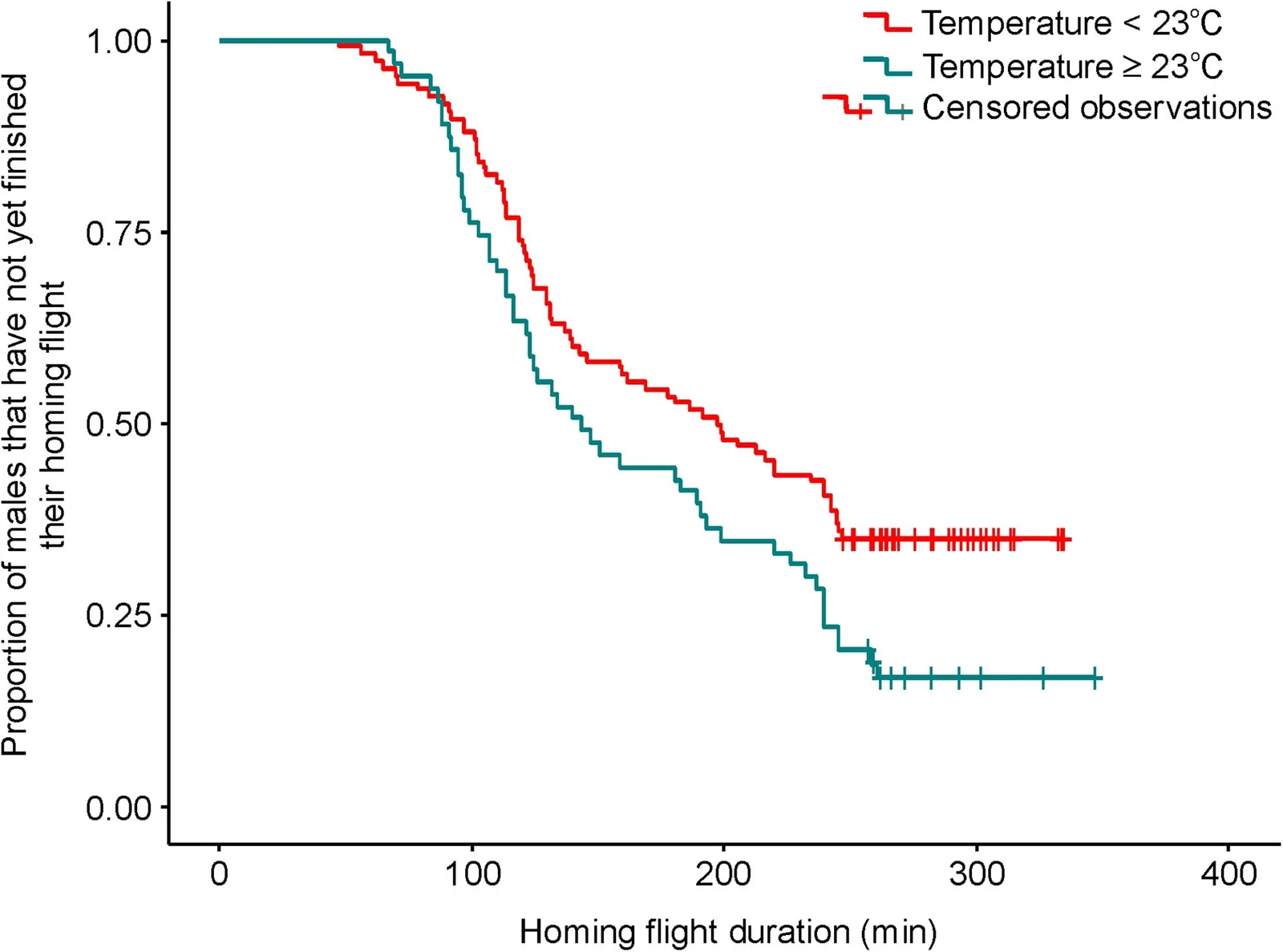
Survival curves illustrating the relationship between the mean air temperature during the homing trial and return efficiency in male barn swallows. The survival curves were compared for two categories of mean temperature: 23 °C and above, and below 23 °C. Lower survival curve indicates faster completion times for the homing flight.

**Table 2:** Results of the final mixed-effects cox proportional hazard model—incorporating male ID as a random intercept—that analyzed the effects of “age” group, “repeat type” category (homing flight repeated “within year” or repeated “among year”), and mean temperature on the hazard of early return from homing flight in male barn swallows.

Fixed effects	Beta ± SE	HR	95% cI	*Z*	*P*
Age:2y-old males	1.387 ± 0.385	4.002	1.883 to 8.508	3.60	<0.001
Age: older males	1.879 ± 0.460	6.547	2.659 to 16.116	4.09	<0.001
Repeat type: within year	1.647 ± 0.252	5.193	3.169 to 8.509	6.54	<0.001
Repeat type: among year	1.848 ± 0.433	6.346	2.718 to 14.818	4.27	<0.001
Mean temperature	0.275 ± 0.137	1.317	1.008 to 1.721	2.02	0.044
**Random effect**	**Variance ± SE**	**95% CI**	**χ^2^**	**df**	***P***
Male: intercept	1.934 ± 0.140	1.485 to 2.623	266.51	66.9	< 0.001
**Model fit**	**Value ± SE**	—	—	—	
Concordance index	0.893 ± 0.020	—	—	—	

Reported are: regression coefficient (beta) with standard error (SE), hazard ratio (HR) with 95% confidence interval (CI), variance for the random intercept of male ID with SE and 95% CI, significance, and model concordance with SE. A HR > 1 indicates an increased hazard of earlier return from homing flight. Concordance represents the proportion of all usable pairs of males where predictions and observed outcomes are concordant, thus measuring the model's discriminative ability (values closer to 1 indicate better prediction).

Seasonal reproductive success did not differ between males that participated in homing trials and untested individuals (*n* = 61), indicating no effect of the experimental treatment: estimate (± SE) = 0.125 (± 0.077), *Z* = 1.624, *P* = 0.104.

## Discussion

Through homing experiments, I investigated whether the homing performance of male barn swallows changes with age or with experience gained from a previous homing flight along the same route, either in the same year or a previous one. I found that the likelihood of males returning early from a displacement site to the breeding territory—a measure of homing performance—consistently and significantly increases with male age. The homing performance showed significant but modest repeatability among male barn swallows—some males consistently achieved shorter return times, while others consistently recorded longer ones. Second, I found that homing flights from the same release location were significantly faster on the second attempt than on the first, regardless of whether they occurred in the same year or the following year. Moreover, higher mean temperatures during the homing trial were significantly associated with an increased likelihood of early arrival from the homing flight.

All male barn swallows returned from the homing flight (homing rate was 100% in a sample of 171 homing trials conducted on 97 males), but substantial differences in homing performance were observed, influenced by two a priori predictors, both of which appeared robust and significant. Older males homed faster than 2-y-old males, and both of these age groups outperformed 1y-olds (Tables 1 and 2, Fig. 1). These findings demonstrate that the homing performance of adult migratory birds with prior migratory experience increases with age, providing the first empirical support for a relationship that could have been anticipated, but had not previously been tested. All of the males that took part in this study experienced at least one episode of migration to and from Africa.

In earlier studies on homing performance in barn swallows, Wojtusiak et al. (1980) tested both juvenile and adult birds. They showed that experienced adult birds had a higher likelihood of returning to the breeding colony compared with juveniles. However, the very small sample size precluded robust statistical evaluation of these differences and prevents drawing reliable conclusions in this case. Studies on homing pigeons have shown that the homing ability—defined as route efficiency—is significantly higher in several year old, experienced birds than in juveniles or 1y-old, inexperienced birds (Schiffner and Wiltschko 2013; Griffiths et al. 2021). Interestingly, this difference disappeared after several repeated homing flights from the same release site. Griffiths et al. (2021) concluded that older, experienced pigeons make more effective use of visual landmarks when navigating home, whereas young, inexperienced birds engage in more exploratory behavior during the return flight. However, within the cohort of experienced, several-year-old pigeons, homing efficiency showed only a negligible increase with age (Schiffner and Wiltschko 2013).

In their research on migratory performance in in black kites, Sergio et al. (2014) found that several aspects of migration efficiency—including route efficiency and migration speed—improve with age. This enhancement in migratory performance across consecutive age groups was attributed partly to individual improvement and partly to the selective mortality of inferior performers. In the present study, the improvement in individual birds’ ability to return to their nests quickly was confirmed through comparisons of homing times recorded for the same barn swallow males over two consecutive years. However, the possibility that selective mortality also contributes to variation in homing efficiency among age classes of males cannot be ruled out. Future studies could investigate the relationship between homing performance and annual survival prospects in wild birds. Another potentially interesting direction for future research is to explore whether individuals with superior homing abilities are also more efficient migrants in migratory bird species.

Within-individual improvement in navigational skills is one factor that may explain the increase in homing performance with age. However, this issue lies beyond the scope of the present study due to the challenges associated with fine-scale tracking of small passerine birds. Another contributing factor is the motivation to return to the breeding territory quickly. Mating status, reproductive condition, or mating opportunities are among the factors that may motivate displaced individuals to rush home (Mehlhorn and Rehkaemper 2016; Zhang et al. 2024). Lifjeld et al. (2022) reported that both within-pair and extra-pair fertilization success increased gradually up to the age of four in male barn swallows. The increase in fertilization success was most pronounced between 1y- and 2y-old males. The increase in reproductive effort with age in male barn swallows is also evident in progressively earlier spring arrival times and earlier breeding dates during the first 3–4 years of life, the former likely resulting from improved migratory performance (Møller 1994; Møller and de Lope 1999; Matyjasiak 2013; Lifjeld et al. 2022). If the age-related improvement in homing performance observed in this study is driven by higher reproductive effort and success in older males, one would expect individual males’ homing speed to increase with their fertilization success, fecundity and/or seasonal reproductive success. Furthermore, it would be interesting to compare homing performance between male and female barn swallows across different age groups. Generally, female birds have greater certainty of their maternity than males do of their paternity, although intraspecific brood parasitism may complicate the relationship (Petrželková et al. 2015). One might expect that improvement in homing performance with age will be less pronounced in females than in males. These issues warrant investigation in future studies.

I found that the return time was significantly shorter during the second homing flight from the same release location—whether in the same year or the following year—than during the first (Table 2; Fig. 2). This supports the hypothesis that experience and route knowledge may help reduce homing time. This result is consistent with findings from previous studies on birds, primarily homing pigeons (Meade et al. 2005; Martinho et al. 2015; Pettit et al. 2015; Mehlhorn and Rehkaemper 2016; Taylor et al. 2017), but also hirundines—barn swallows (Wojtusiak and Ferens 1947; Wojtusiak et al. 1979a, 1980) and sand martins *Riparia riparia* (Giunchi et al. 1999). A more advanced breeding stage as a motivating factor for faster homing during the second flight can be ruled out as an explanation for this pattern, because model screening showed that this factor did not significantly contribute to explaining return time (Supplementary Table S1). However, the improvement in homing performance may be driven by birds learning visual cues, such as natural or human-made landmarks, at the release site and in the area they subsequently overfly. Another potentially influential factor may be the familiarity gained during the first homing flight with key locations—such as local breeding colonies or potential feeding areas—situated at the release site and along the homing route. Familiarity with these key locations can reduce homing time on the second flight. In this study, the release site was located beyond the typical foraging range of barn swallows residing in breeding colonies, yet within the species’ natal dispersal distance (see Study species and site section, above). Therefore, it cannot be ruled out that some focal males were already familiar with the site from prior experience. Nevertheless, the observed improvement in homing performance on the second flight highlights the substantial value of the experience gained during the first flight. If lightweight tracking devices suitable for monitoring small passerines during long-distance flights become available, future research could examine how experience influences the route efficiency in barn swallows.

The homing performance of male barn swallows was not associated with either their flight morphology or body size and condition (Supplementary Table S1). Of the weather factors considered, only mean air temperature had a positive effect on the hazard of an early return from a homing flight. Higher ambient temperatures can improve foraging opportunities (Saino et al 2004; Martay et al. 2023) and possibly promote faster return from a homing flight. The wind speed and direction did not affect the results; however, it should be noted that the homing experiments were conducted on sunny days without high winds or precipitation. Previous studies on barn swallow homing behavior have shown that homing success is significantly lower on foggy, cool, windy, and rainy days (Wojtusiak et al. 1979a, 1979b, 1980).

Summing up, the results of the present study support the hypothesis that the likelihood of an early return from a homing flight increases with age and experience gained from previous flights from the same release site in male barn swallows. These findings are important because they show that, in adult migratory birds, homing performance improves progressively with age and accumulated experience. I highlight the lack of comprehensive research on the factors influencing homing performance in seasonally migratory bird species across diverse taxonomic groups. In this context, further progress in developing lightweight tracking devices suitable for precisely monitoring small passerines during long-distance flights will be essential for advancing future research on homing ability in these birds.

## Supplementary Material

zoaf080_Supplementary_Data

## References

[zoaf080-B1] Able KP , 1991. The development of migratory orientation mechanisms. In: Berthold P, editors. Orientation in Birds. Basel: Birkhäuser Verlag, 166–179. 10.1007/978-3-0348-7208-9_8 1838514

[zoaf080-B2] Able KP , 2001. The concepts and terminology of bird navigation. J Avian Biol 32(2):174–183.

[zoaf080-B3] Åkesson S , 2003. Avian long-distance navigation: experiments with migratory birds. In: Berthold P, Gwinner E, Sonnenschein E, editors. Avian Migration. Berlin Heidelberg: Springer-Verlag, 471–492.

[zoaf080-B4] Åkesson S, Bakam H, Martinez Hernandez E, Ilieva M, Bianco G, 2021. Migratory orientation in inexperienced and experienced avian migrants. Ethol Ecol Evol 33(3):206–229.

[zoaf080-B5] Åkesson S, Boström J, Liedvogel M, Muheim R, 2014. Animal navigation. In: Hansson L-A, Åkesson S, editors. Animal Movement Across Scales. Oxford: Oxford University Press, 151–178.

[zoaf080-B6] Åkesson S, Hedenström A, 2007. How migrants get there: migratory performance and orientation. BioScience 57(2):123–133.

[zoaf080-B7] Biro D, Sumpter DJ, Meade J, Guilford T, 2006. From compromise to leadership in pigeon homing. Curr Biol 16(21):2123–2128. 10.1016/j.cub.2006.08.087 17084696

[zoaf080-B8] Blaser N, Dell'Omo G, Dell'Ariccia G, Wolfer DP, Lipp HP, 2013. Testing cognitive navigation in unknown territories: homing pigeons choose different targets. J Exp Biol 216(16):3123–3131. 10.1242/jeb.083246 23885090

[zoaf080-B9] Burnham KP, Anderson DR, 2002. Model Selection and Multimodel Inference: A Practical Information-Theoretic Approach. New York: Springer-Verlag Inc.

[zoaf080-B10] Dell'Ariccia G, Costantini D, Dell'Omo G, Lipp HP, 2009. Waiting time before release increases the motivation to home in homing pigeons (*Columba livia*). J Exp Biol 212(20):3361–3364. 10.1242/jeb.032995 19801440

[zoaf080-B11] Dell'Ariccia G, Dell'Omo G, Wolfer DP, Lipp HP, 2008. Flock flying improves pigeons’ homing: GPS track analysis of individual flyers versus small groups. Anim Behav 76(4):1165–1172.

[zoaf080-B12] Fox J, Weisberg S, 2019. An R Companion to Applied Regression. 3rd edn. Los Angeles: Sage Publications.

[zoaf080-B13] Giunchi D, Mongini E, Baldaccini NE, 1999. The influence of repeated releases on the homing behaviour of sand martins (*Riparia riparia*). Ethology 105(2):111–124.

[zoaf080-B14] Griffiths C, Schiffner I, Price E, Charnell-Hughes M, Kishkinev D et al, 2021. Repeated training of homing pigeons reveals age-dependent idiosyncrasy and visual landmark use. Anim Behav 177:159–170.

[zoaf080-B15] Guilford T, Biro D, 2014. Route following and the pigeon's familiar area map. J Exp Biol 217(2):169–179. 10.1242/jeb.092908 24431141

[zoaf080-B16] Harrison XA, Donaldson L, Correa-Cano ME, Evans J, Fisher DN et al, 2018. A brief introduction to mixed effects modelling and multi-model inference in ecology. PeerJ 6:e4794. 10.7717/peerj.4794 PMC5970551 29844961

[zoaf080-B17] Jesmer BR, Merkle JA, Goheen JR, Aikens EO, Beck JL et al, 2018. Is ungulate migration culturally transmitted? Evidence of social learning from translocated animals. Science 361(6406):1023–1025. 10.1126/science.aat0985 30190405

[zoaf080-B18] Lewis MA, Fagan WF, Auger-Méthé M, Frair J, Fryxell JM et al, 2021. Learning and animal movement. Front Ecol Evol 9:681704.

[zoaf080-B19] Lifjeld JT, Kleven O, Fossøy F, Jacobsen F, Laskemoen T et al, 2022. When older males sire more offspring—increased attractiveness or higher fertility? Behav Ecol Sociobiol 76(5):61. 10.1007/s00265-022-03170-0 PMC9034975 35535127

[zoaf080-B20] Mandal S , 2018. How do animals find their way back home? A brief overview of homing behavior with special reference to social Hymenoptera. Insectes Soc 65(4):521–536.

[zoaf080-B21] Mann RP, Armstrong C, Meade J, Freeman R, Biro D et al, 2014. Landscape complexity influences route memory formation in navigating pigeons. Biol Lett 10(1):20130885. 10.1098/rsbl.2013.0885 PMC3917332 24451267

[zoaf080-B22] Martay B, Leech DI, Shortall CR, Bell JR, Thackeray SJ et al, 2023. Aerial insect biomass, but not phenological mismatch, is associated with chick survival of an insectivorous bird. Ibis 165(3):790–807.

[zoaf080-B23] Martinho A III, Biro D, Guilford T, Gagliardo A, Kacelnik A, 2015. Asymmetric visual input and route recapitulation in homing pigeons. Proc Biol Sci 282(1816):20151957. 10.1098/rspb.2015.1957 PMC4614786 26446810

[zoaf080-B24] Matyjasiak P , 2013. Timing of arrival from spring migration is associated with flight performance in the migratory barn swallow. Behav Ecol Sociobiol 67(1):91–100. 10.1007/s00265-012-1429-x PMC3536968 23293424

[zoaf080-B25] Matyjasiak P, Chacińska P, Książka P, 2024. Anthropogenic noise interacts with the predation risk assessment in a free-ranging bird. Curr Zool 70(3):277–283. 10.1093/cz/zoad019 PMC11255998 39035757

[zoaf080-B26] Matyjasiak P, López-Calderón C, Ambrosini R, Balbontín J, Costanzo A et al, 2023. Wing morphology covaries with migration distance in a highly aerial insectivorous songbird. Curr Zool 69(3):255–263. 10.1093/cz/zoac044 PMC10284055 37351297

[zoaf080-B27] Matyjasiak P, Olejniczak I, Boniecki P, Møller AP, 2013. Wing characteristics and spring arrival date in Barn Swallows Hirundo rustica. Acta Orn 48(1):81–92.

[zoaf080-B28] Meade J, Biro D, Guilford T, 2005. Homing pigeons develop local route stereotypy. Proc Biol Sci 272(1558):17–23. 10.1098/rspb.2004.2873 PMC1634935 15875565

[zoaf080-B29] Mehlhorn J, Rehkaemper G, 2016. The influence of social parameters on the homing behavior of pigeons. PLoS One 11(11):e0166572. 10.1371/journal.pone.0166572 PMC5112789 27846262

[zoaf080-B30] Møller AP , 1994. Sexual Selection and the Barn Swallow. Oxford: Oxford University Press.

[zoaf080-B31] Møller AP, de Lope F, 1999. Senescence in a short-lived migratory bird: age-dependent morphology, migration, reproduction and parasitism. J Anim Ecol 68(1):163–171.

[zoaf080-B32] Nastase AJ , 1982. Orientation and homing ability of the Barn Swallow. J Field Ornithol 53:15–21.

[zoaf080-B33] Papi F , 1992. Animal Homing. London: Chapman & Hall.

[zoaf080-B34] Pašukonis A, Loretto MC, Landler L, Ringler M, Hödl W, 2014. Homing trajectories and initial orientation in a Neotropical territorial frog, *Allobates femoralis* (Dendrobatidae). Front Zool 11(1):29. 10.1186/1742-9994-11-29 PMC3974440 24666825

[zoaf080-B35] Petrželková A, Michálková R, Albrechtová J, Cepák J, Honza M et al, 2015. Brood parasitism and quasi-parasitism in the European barn swallow *Hirundo rustica rustica*. Behav Ecol Sociobiol 69(9):1405–1414.

[zoaf080-B36] Pettit B, Akos Z, Vicsek T, Biro D, 2015. Speed determines leadership and leadership determines learning during pigeon flocking. Curr Biol 25(23):3132–3137. 10.1016/j.cub.2015.10.044 26628007

[zoaf080-B37] R Core Team , 2024. R: A Language and Environment for Statistical Computing. Vienna, Austria: R Foundation for Statistical Computing [cited 2025 February 6]. http://www.R-project.org/.

[zoaf080-B38] Saino N, Romano M, Ambrosini R, Ferrari RP, Møller AP, 2004. Timing of reproduction and egg quality covary with temperature in the insectivorous Barn Swallow, *Hirundo rustica*. Funct Ecol 18(1):50–57.

[zoaf080-B39] Schiffner I, Wiltschko R, 2013. Development of the navigational system in homing pigeons: increase in complexity of the navigational map. J Exp Biol. 216(14):2675–2681. 10.1242/jeb.085662 23580726

[zoaf080-B40] Sergio F, Tanferna A, De Stephanis R, Jiménez LL, Blas J et al, 2014. Individual improvements and selective mortality shape lifelong migratory performance. Nature 515(7527):410–413. 10.1038/nature13696 25252973

[zoaf080-B41] Silovský V, Landler L, Faltusová M, Börger L, Burda H et al, 2024. A GPS assisted translocation experiment to study the homing behavior of red deer. Sci Rep 14(1):6770. 10.1038/s41598-024-56951-0 PMC10958021 38514686

[zoaf080-B42] Steiner I, Bürgi C, Werffeli S, Dell'Omo G, Valenti P et al, 2000. A GPS logger and software for analysis of homing in pigeons and small mammals. Physiol Behav 71(5):589–596. 10.1016/s0031-9384(00)00409-1 11239679

[zoaf080-B43] Stoffel MA, Nakagawa S, Schielzeth H, 2017. Rptr: repeatability estimation and variance decomposition by generalized linear mixed-effects models. Methods Ecol Evol 8(11):1639–1644.

[zoaf080-B44] Svensson L , 1992. Identification Guide to European Passerines. 4th edn. Stockholm: British Trust for Ornithology.

[zoaf080-B45] Taylor LA, Portugal SJ, Biro D, 2017. Homing pigeons (*Columba livia*) modulate wingbeat characteristics as a function of route familiarity. J Exp Biol 220(16):2908–2915. 10.1242/jeb.154039 28814611

[zoaf080-B46] Therneau TM , 2024a. A Package for Survival Analysis in R. R package version 3.8-3 [cited 2025 January 30]. Available from: https://CRAN.R-project.org/package=survival.

[zoaf080-B47] Therneau TM , 2024b. Coxme: mixed effects Cox models. R package version 2.2-22. 2024 [cited 2024 October 20]. Available from: https://CRAN.R-project.org/package=coxme.

[zoaf080-B48] Turner A , 2006. The Barn Swallow. London: T&AD Poyser.

[zoaf080-B49] Wallraff HG , 2004. Avian olfactory navigation: its empirical foundation and conceptual state. Anim Behav 67(2):189–204.

[zoaf080-B50] Ward AJW, James R, Wilson ADM, Webster MM, 2013. Site fidelity and localised homing behaviour in three-spined sticklebacks (*Gasterosteus aculeatus*). Behaviour 150(14):1689–1708.

[zoaf080-B51] Williams HJ, Safi K, 2021. Certainty and integration of options in animal movement. Trends Ecol Evol 36(11):990–999. 10.1016/j.tree.2021.06.013 34303526

[zoaf080-B52] Wiltschko R , 1991. The role of experience in avian navigation and homing. In: Berthold P, editor. Orientation in birds. Basel: Birkhäuser Verlag, 250–269. 10.1007/978-3-0348-7208-9_12 1838518

[zoaf080-B53] Wiltschko R, Wiltschko W, 2015. Avian navigation: a combination of innate and learned mechanisms. Adv Study Behav 47:229–310.

[zoaf080-B54] Wojtusiak RJ , 1949. Polish investigations on homing in birds and their orientation in space. Proc Linn Soc London 160(2):99–108.

[zoaf080-B55] Wojtusiak RJ, Bocheński Z, Dylewska M, Gieszczykiewicz J, 1979a. Homing experiments on birds. XII. Influence of training and transport direction on homeward orientation of swallows *Hirundo rustica* L. Folia Biol 27:269–276. 535636

[zoaf080-B56] Wojtusiak RJ, Bocheński Z, Harmata W, Majlert Z, 1979b. Homing experiments on birds. XIV. The influence of the route of transportation on the speed of return in the swallow (*Hirundo rustica* L.). Folia Biol 27:169–179. 499608

[zoaf080-B57] Wojtusiak RJ, Ferens B, 1947. Homing experiments on birds. VII. Further investigations on the velocity of swallows (*Hirundo rustica* L.) and on the role of memory in their orientation in space. Bull Int Acad Polon Sc. Cracov. B II:135–164.

[zoaf080-B58] Wojtusiak RJ, Majlert Z, Franckiewicz H, Harmata W, 1980. Homing experiments on birds. Part XV. The influence of radio waves on the homing of swallows (*Hirundo rustica* L.). Folia Biol 28:127–144. 7428978

[zoaf080-B59] Zannoni N, Wikelski M, Gagliardo A, Raza A, Kramer S et al, 2020. Identifying volatile organic compounds used for olfactory navigation by homing pigeons. Sci Rep 10(1):15879. 10.1038/s41598-020-72525-2 PMC7523013 32985543

[zoaf080-B60] Zhang YD, Chou CC, Liao CP, Lin JW, Clark RW et al, 2024. Homing behavior of a tree lizard: influences of mating resource and habitat structure. Behav Ecol 35(6):arae081.

[zoaf080-B61] Zuur AF, Ieno EN, Walker NJ, Saveliev AA, Smith GM, 2009. Mixed Effects Models and Extensions in Ecology with R. New York: Springer.

