# Supplementary material for: Age, experience, and homing performance in a long-distance migrating bird, the barn swallow
Source: Curr Zool. 2025 Dec 22;72(5):714–22. doi: 10.1093/cz/zoaf080 (PMC13690572; doi:10.1093/cz/zoaf080)
Supplement: Supplementary file 1 [file zoaf080_supplementary_data.pdf]

Supplementary online material for

Age, experience, and homing performance in a long-distance migrating bird, the barn swallow

by

Piotr MATYJASIAK

Contents:

Supplementary Table S1 ……… page 2-3

Supplementary Table S2 ……… page 4

Supplementary Table S3 ……… page 5

Supplementary Figure S1 ……… page 6

Supplementary Table S1. Screening results from the mixed-effects Cox proportional hazards models that tested the effect of the additional predictors on the homing flight completion time. The analysis began with a null model that included only the random structure (1 | colony/male). This was followed by an initial model that included two *a priori* predictors: 'age' (3-level factor; '1y-old' [reference], '2y-old', and 'older' males) and 'repeat type' (3-level factor: 'first flight' [reference], 'within year', and 'among year'). Then, screening models were constructed by adding one additional predictor to the initial model at a time. One model tested the interaction between 'age' and 'breeding stage'. The Table reports the following for each model: the model structure, the maximized log-likelihood of the model (*LL*); the number of estimated parameters (*k*); and the model selection metrics, which include the small-sample corrected Akaike Information Criterion (AICc) and the ΔAICc relative to the initial model (the calculation methods for AIC and AICc are provided in the table footer). Predictors that met the inclusion criterion (ΔAICc ≤ −2 relative to the initial model) in the final Cox mixed-effects model are shown in **bold**.

**Model**	**Structure**	***LL***	***K***	**AICc[(a)]**	**ΔAICc vs. initial model**
Null	~ (1|colony/male)	-566.1	2	1136.3	76.9
Initial (predictors *a priori*)	~ age + repeat_type + (1|colony/male)	-523.4	6	1059.3	0.0
Breeding stage	~ age + repeat_type + breeding_stage + (1|colony/male)	-523.2	7	1061.1	1.8
Interaction age * breeding stage	~ repeat_type + age * breeding_stage + (1|colony/male)	-523.1	9	1065.3	5.9
Release order	~ age + repeat_type + release_order + (1|colony/male)	-522.6	7	1059.9	0.6
	Flight morphology and body size variables				
Keel length	~ age + repeat_type + keel + (1|colony/male)	-523.2	7	1061.0	1.6
Wing aspect ratio	~ age + repeat_type + aspect_ratio + (1|colony/male)	-523.4	7	1061.5	2.2
Tail length	~ age + repeat_type + tail + (1|colony/male)	-522.5	7	1059.6	0.3
Body weight	~ age + repeat_type + body_weight + (1|colony/male)	-523.1	7	1060.9	1.6
	Weather conditions during homing trial				
**Mean temperature[(b)]**	**~ age + repeat_type + temperature + (1|colony/male)**	**-521.3**	**7**	**1057.3**	**-2.1**
Wind speed	~ age + repeat_type + wind_speed + (1|colony/male)	-523.4	7	1061.5	2.2
Dominant wind direction	~ age + repeat_type + wind_direction + (1|colony/male)	-523.4	7	1061.5	2.1

(a) For ‘coxme’ models, the Akaike Information Criterion values were computed manually as AIC = $-2 \cdot LL + 2 \cdot k$, where the number of estimated parameters, k, was counted as the number of fixed-effect coefficients (including contrasts) plus the variance parameters for random effects. For the nested random structure (1 | colony/male), two variance parameters were counted (one for the colony and one for the male within the colony). AICc values were computed as AICc = AIC + $(2 \cdot k \cdot (k + 1)) / (n - k - 1)$, where n is the number of observations (data rows). The same dataset was used for all models.

(b) Since only ‘mean temperature’ met the inclusion criterion for the final model, the screening model containing ‘mean temperature’ became the final model.

Supplementary Table S2. Results of the initial Cox proportional hazards model with frailty term for male ID. The model examines the effects of two *a priori* predictors: 'age' group and 'repeat type' category (homing flight repeated 'within year' or repeated 'among year') on the time to complete homing flight. Reported are: regression coefficient (beta) with standard error (SE), hazard ratio (HR) with 95% confidence interval (CI), significance, frailty variance (theta) for the random intercept of male ID with SE and 95% CI, 95% CI, and model concordance with SE. Concordance represents the proportion of all usable pairs of males where predictions and observed outcomes are concordant, thus measuring the model's discriminative ability (values closer to 1 indicate better prediction). A HR > 1 indicates an increased hazard of earlier return from homing flight.

**Fixed effects**	**Beta ± SE**	**HR**	**95% CI**	χ^2	**df**	***P***
Age:2y-old males	1.201 ± 0.314	3.325	1.795 to 6.157	14.60	1	< 0.001
Age: older males	1.487 ± 0.347	4.422	2.239 to 8.732	18.34	1	< 0.001
Repeat type: within year	1.405 ± 0.233	4.077	2.585 to 6.432	36.52	1	< 0.001
Repeat type: among year	1.523 ± 0.367	4.584	2.233 to 9.412	17.21	1	< 0.001
**Random intercept (frailty term)**	**Theta ± SE**		**95% CI**	χ^2	**df**	***P***
Male (frailty)	0.833 ± 0.364		0.118 to 1.547	67.71	38.6	0.003
**Model fit**	**Value ± SE**					
Concordance index	0.834 ± 0.017					

Supplementary Table S3. Results of the final Cox proportional hazards model with frailty term for male ID. The model examines the effects of ‘age’ group and ‘repeat type’ category (homing flight repeated ‘within year’ or repeated ‘among year’), and mean temperature on the time to complete homing flight. Reported are: regression coefficient (beta) with standard error (SE), hazard ratio (HR) with 95% confidence interval (CI), significance, frailty variance (theta) for the random intercept of male ID with SE and 95% CI, and model concordance with SE. Concordance represents the proportion of all usable pairs of males where predictions and observed outcomes are concordant, thus measuring the model's discriminative ability (values closer to 1 indicate better prediction). A HR > 1 indicates an increased hazard of earlier return from homing flight.

**Fixed effects**	**Beta ± SE**	**HR**	**95% CI**	χ^2	**Df**	***P***
Age:2y-old males	1.218 ± 0.320	3.380	1.806 to 6.325	14.50	1	< 0.001
Age: older males	1.651 ± 0.360	5.214	2.575 to 10.559	21.04	1	< 0.001
Repeat type: within year	1.401 ± 0.233	4.061	2.573 to 6.410	36.22	1	< 0.001
Repeat type: among year	1.397 ± 0.379	4.042	1.923 to 8.496	13.58	1	< 0.001
Mean temperature	0.245 ± 0.120	1.278	1.010 to 1.616	4.19	1	0.041
**Random effect (frailty term)**	**Theta ± SE**		**95% CI**	χ^2	**Df**	***P***
Male (frailty)	0.877 ± 0.368		0.155 to 1.598	71.28	39.3	0.001
**Model fit**	**Value ± SE**					
Concordance index	0.836 ± 0.016					

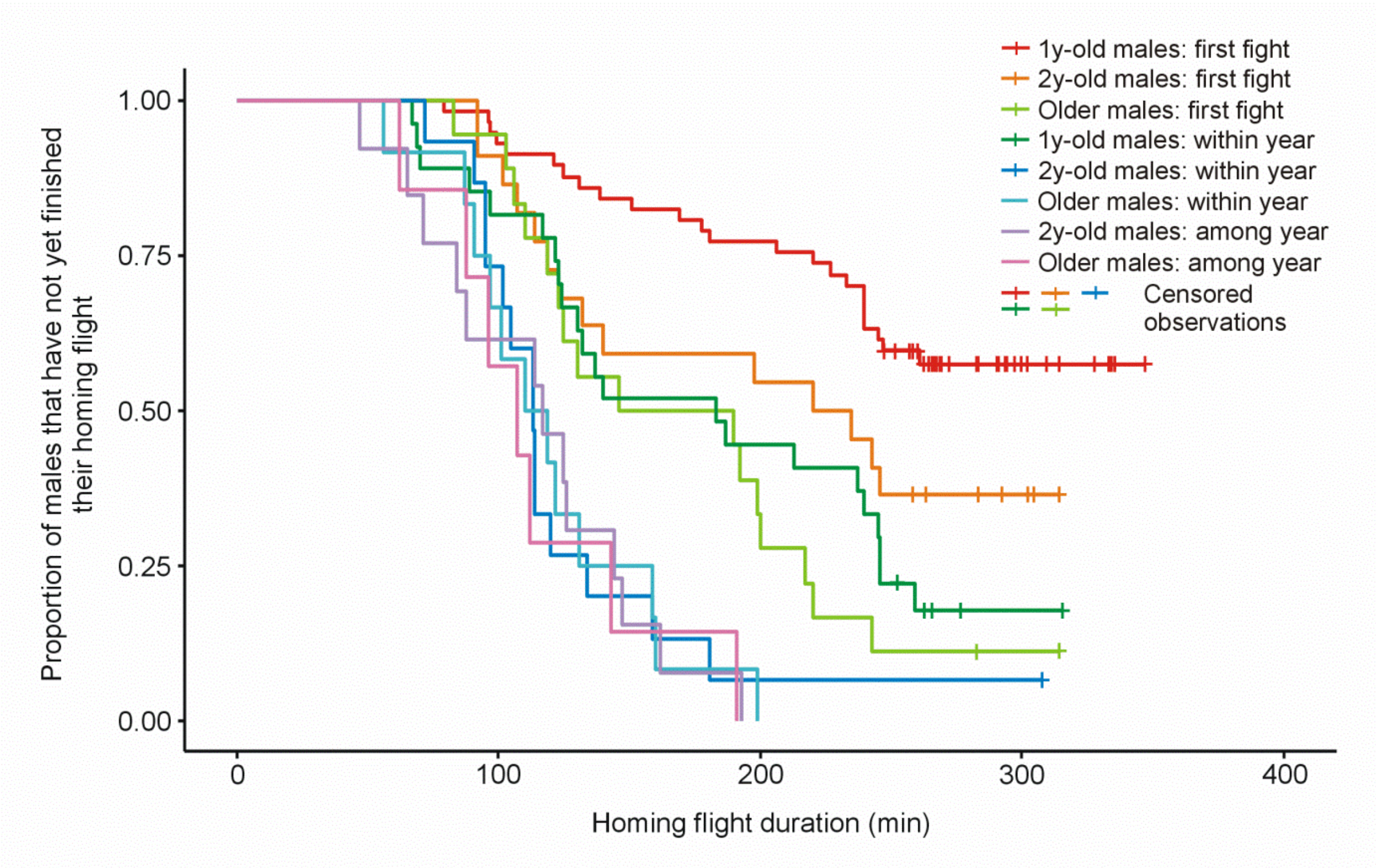

Supplementary Figure S1. Survival curves illustrating the probability of continuing the homing flight as a function of male barn swallows' age group and homing trial. The survival curves were estimated separately for each combination of 'age' category ('2y-old' males vs. '3y-old' and 'older' males) and homing trial ('repeat type'; 'first flight' vs. homing flight repeated 'within-year' or repeated 'among-year'). Lower survival curves indicate faster completion times for the homing flight. As shown in the plot, older males and those participating in the second homing trial tended to complete the homing flight more quickly. This is reflected by the steeper decline in survival probability over time. These homing trials could be repeated from the same release site in either the same year ('within year') or the following year ('among year').
